# Improved Fault Diagnosis in Hydraulic Systems with Gated Convolutional Autoencoder and Partially Simulated Data

**DOI:** 10.3390/s21134410

**Published:** 2021-06-27

**Authors:** Albert Gareev, Vladimir Protsenko, Dmitriy Stadnik, Pavel Greshniakov, Yuriy Yuzifovich, Evgeniy Minaev, Asgat Gimadiev, Artem Nikonorov

**Affiliations:** 1 Samara National Research University Named after S.P. Korolev, Moskovskoye Shosse 34, 443086 Samara, Russia; gareevalbert@mail.ru (A.G.); protsenkovi@ssau.com (V.P.); pavel.ssau@gmail.com (P.G.); yuriyvyuzifovich@gmail.com (Y.Y.); gimadiev_ag@mail.ru (A.G.); artniko@gmail.com (A.N.); 2 Image Processing Systems Institute of the RAS—Branch of the Federal Scientific Research Centre “Crystallography and Photonics” of the Russian Academy of Sciences, Molodogvardeyskaya 151, 443001 Samara, Russia

**Keywords:** intelligent fault detection, hydraulic systems, sensor signals, classification

## Abstract

This paper examines the effectiveness of neural network algorithms for hydraulic system fault detection and a novel neural network architecture is suggested. The proposed gated convolutional autoencoder was trained on a simulated training set augmented with just 0.2% data from the real test bench, dramatically reducing the time needed to spend with the actual hardware to build a high-quality fault detection model. Our fault detection model was validated on a test bench and showed accuracy of more than 99% of correctly recognized hydraulic system states with a 10-s sampling window. This model can be also leveraged to examine the decision boundaries of the classifier in the two-dimensional embedding space.

## 1. Introduction

Maintenance represents a significant portion of the manufacturing costs, ranging from 15% to 70% [1,2,3], depending on the industry, a well-known fact confirmed by numerous studies [4,5,6,7]. For the technology infrastructure, losses from the downtime can be quite significant, with hourly loss ranging from hundreds of thousands to millions of US dollars [8,9]. Maintenance best practices have been evolving from reactive maintenance to preventive maintenance to predictive maintenance [2], with intelligent fault diagnosis (IFD), using recent advances in industrial Internet of Things, artificial intelligence, edge computing [10,11], etc.

Many studies examine system monitoring and suggest various mathematical models and methods for fault detection [12,13,14,15]. Papers [12,13] consider model-based and DSP-based fault prediction, while papers [14,15] and more recent ones use data-driven approaches. Data-driven and deep learning-based methods show great results not only in computer vision applications [16,17], speech recognition [18], natural language processing [19], and medical imaging [20,21], but also as classifiers for induction motor fault classification [22], railway vehicle wheels diagnosis [23], industrial machinery [24], hydraulic system malfunction identification [25], and fault diagnosis of aircraft engines [26]. A comprehensive review of intelligent fault diagnostic methods presented as a single timeline is provided in [27].

Most of the papers address specific IFD algorithms and classifiers. Vibration data [28,29] and statistical and heuristic feature engineering performed with this data [30,31] is typically used as classifier input, while works [10,32] use additional sensors such as temperature, pressure, and volume flow. Authors of [31] provided a comprehensive list and experimental evaluation of the most modern data-driven IFD algorithms, with the source code available. Since data collection and preprocessing phases are critical for IFD development and deployment [2], simulated or synthetic data sets can reduce IFD development complexity and costs. Authors of [33] propose using digital twins to generate datasets to train IFD classifiers attempting to closely match the real environment. Unfortunately, using simulated data and transferring the learning across systems may not be the perfect solution as collected data may have different distributions [29], needs domain adaptation [34] and can suffer from class imbalances [2]. All these problems lead to the less accurate IFD on the real data. The search for new dataset collection methods and classifiers that allow the usage of inexpensive simulated data for training without introducing a prediction accuracy penalty, remains an important research question.

In this paper, we propose a method to collect a training dataset and a new classifier model that uses both simulated and obtained from the physical system data. Our physical system was simulated with Simulation X software [35,36].

Our new IFD classifier architecture is based on the gated convolutional autoencoder. The model was trained on the simulated dataset mixed with a small portion (less than 0.2%) of the data collected from the physical system. The inclusion of the real data, even a small amount of it compared to the simulated data, improved IFD classification performance when the trained model was applied to the physical system.

Our gated convolutional autoencoder, trained on the combination of the data from the simulated and physical data, shows 99% average accuracy when applied to the physical system. We open-sourced not only the source code, but also the simulated and physical datasets used in this paper, located at https://gitlab.com/protsenkovi/efd_nn (accessed on 25 June 2021).

## 2. Intelligent Fault Diagnosis Problem Definition

We define an IFD task as a multiclass classification task for N types of faults. The class “zero” corresponds to the healthy system and other classes labeled from 1 to N correspond to specific faults. This reduces the problem of fault diagnosis to the problem of (N + 1)-class classification. In this paper we only considered mutually exclusive faults; multiple faults can be added as additional fault classes.

The classification problem is solved using the data from M sensors installed on the hydraulic system. We assume that all data has a uniform sampling in the time domain. Where it is not the case, interpolation can always be performed. The data with the control action applied to the system is assumed to come from one of the sensors.

Classification for each fault class c starts with a set of sensor measurements of the length W, the window size. We use a fixed-length window, measured in seconds, and the number of samples in the window depends on the sampling rate. In general, the state of the system can be described in the form of a fault class *c*, where zero class corresponds to a healthy system:

(1) c=G(X,a)

where *X* is a W × M data matrix, each column represents the data from an individual sensor, each row represents a single measurement, *a* is a vector of the classifier parameters determined during the training process, and *G* is the decision rule of the classifier that determines the overall state of the system. The decision rule *G* is implemented by the neural network-based classifier described in Section 4. We move a sliding window W over the training data to create the training dataset *X*.

**Figure 1:**
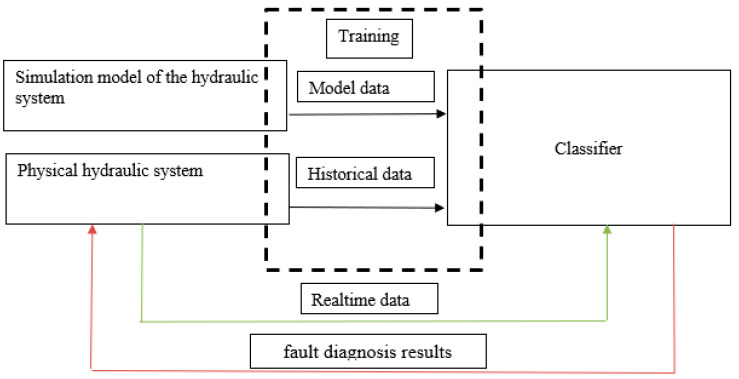
Architecture of the proposed intelligent fault diagnosis system.

To train a neural network classifier, a large set of data for each fault is typically required, which is hard to obtain from any physical hydraulic system for an exhaustive combination of faults and control actions. Instead, we use a simulation model of the system to obtain fault data under various control actions. The combination of the data obtained from the simulation model with a small amount of historical data collected from the physical system is used to train classifiers. The validation of the trained model is performed with the data received from the physical system with a malfunctioning subsystem. The test bench was designed to produce a variety of manually triggered faults. The architecture of the proposed intelligent fault diagnosis system is presented in Figure 1.

Our approach that combines a simulation model of the hydraulic system with the real data, lowered the amount of historical data needed without compromising the flexibility and versatility of the machine learning model. The inclusion of just 0.2% of the physical system data in the training dataset produced the required classification quality.

## 3. Our Physical and Simulated Hydraulic Systems

### 3.1. Our Hydraulic System and Sensor Setup

**Figure 2:**
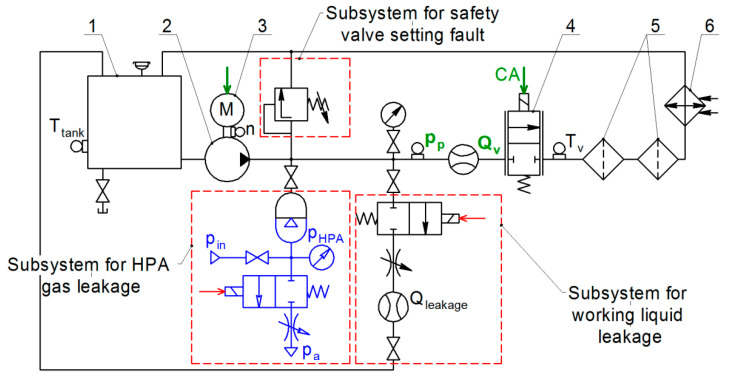
A diagram of a typical hydraulic system (working fluid supply station) modelling the faults: CA—control action; TS—temperature sensor; RS—rotational velocity sensor; PS—pressure sensor; FS—flow rate sensor.

Figure 2 shows a schematics of a typical hydraulic system (HS) that supplies the pressure and the fluid flow to the hydraulic drive. The dashed red line indicates components where the following three malfunctions can occur: gas leak from the hydro-pneumatic accumulator (“HPA gas leakage”); leakage of the working fluid from the pressure line at the outlet of the pump (“Fluid leakage”); safety valve adjustment error (valve setting fault).

Our hydraulic system operates as follows. The pump driven by the electric motor circulates the working fluid through the proportional valve, hydraulic filters, heat exchanger, and back to the tank. The nominal flow rate generated by the pump running at 2500 rpm and a pressure of 40 bar is 32 L/min. The heat exchanger maintains the constant fluid temperature of 50 °C. Hydraulic filters provide the filtration of the working fluid. The proportional valve is used to “load” the system by changing the flow rate in accordance with the control signal applied to its coil.

The control signal in the system is a voltage varying from 2 V to 4 V that follows normal distribution with an average voltage of 3 V and a standard deviation σ = 2/3 with a sampling time of 1.5 s and the constant first order lag of 11 ms. The HPA reduces pump pressure pulsations and increases the capacitance of the system.

The three system faults are implemented as follows. We create gas leakage from the HPA by connecting its gas chamber with the ambient atmosphere. We consider only complete discharge of the HPA as the HPA malfunction state. The amount of leakage of the fluid at the outlet of the pump is set using the throttle that lets some fluid to pass into the tank, bypassing the proportional valve. Average values of leaks characterizing various states of hydraulic system degradation are 2.3 L/min, 3.4 L/min, 4.6 L/min, and 5.8 L/min. We simulate the setting error of the safety valve by changing the preload force of its spring, corresponding to the opening pressures of 36.1 bar, 29.3 bar, 22.3 bar, and 15.2 bar. When the system is operating normally, the opening pressure of the safety valve is 46.5 bar. For each state of the system with the parameters listed above we performed experiments and resulting data sets were recorded. 

**Figure 3:**
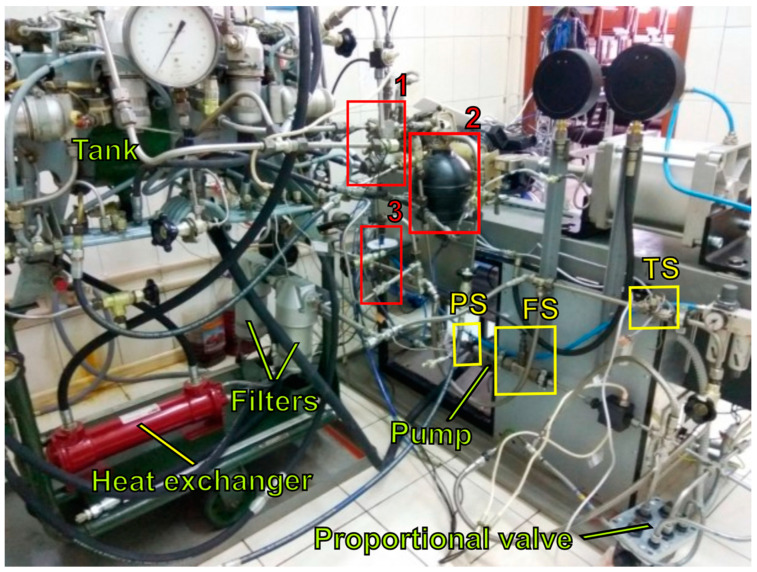
Our bench: 1—subsystem for HPA gas leakage; 2—subsystem for fluid leakage; 3—subsystem for the valve setting fault; PS—pressure sensor; FS—flow sensor; TS—temperature sensor.

There are two monitoring target variables: pump outlet pressure (P_p_) and the proportional valve flow rate (Q_v_). A piezoresistive pressure sensor is installed in the discharge pipeline at a distance of 30 pipe diameters from the pump. A turbine flow sensor is installed downstream (Figure 3). To measure the temperature of the fluid, a thermal resistance sensor is installed behind the proportional valve. The temperature sensor is connected to the temperature control system, which operates a discrete on/off valve that controls the flow of the water to the heat exchanger to cool the fluid. A gas leak from the HPA or a liquid leak at the pump outlet is simulated by opening discrete valves installed in subsystems 1 and 2, in Figure 3. The desired level of the fluid leakage is set by the bypass throttle in the subsystem 2. The opening pressure of the safety valve can be controlled by its spring preload force, located in the subsystem 3.

Before starting the tests, the required state of the system degradation is set by adjusting the corresponding elements of subsystems 1–3. After starting the pump motor, the pressure signals in the pressure line (P_p_) and the proportional valve flow rate (Q_v_) are recorded by the signal processing system with a 5 kHz sampling rate. Each dataset for a specific state of the system is recorded for 200 s. During this time, there are 130 proportional valve interventions. The total number of datasets is 19. The recorded data is saved as an Mx3 matrix with the discharge line pressure and the flow rate data, as well as the control signal.

### 3.2. The Simulation Model

**Figure 4:**
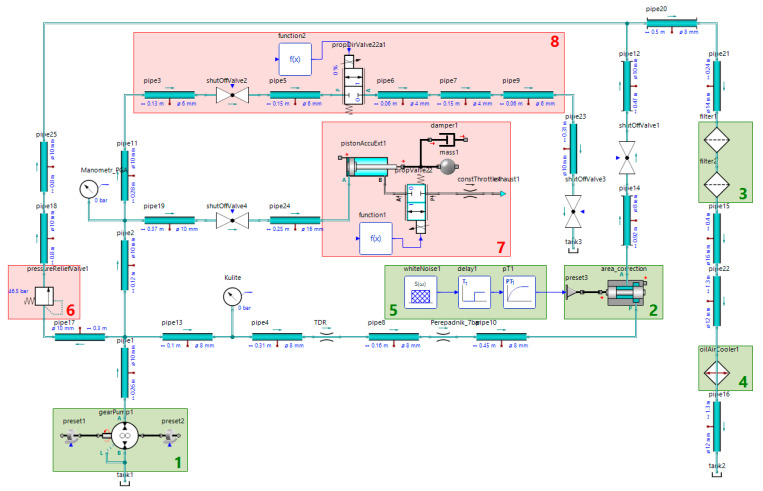
The SimulationX model of our fluid power supply system with built-in faults: 1—the pump with the drive motor; 2—the proportional valve; 3—hydraulic filters; 4—the heat exchanger; 5—the proportional distributor control unit; 6—the unit to simulate safety valve fault; 7—the unit to simulate HPA gas leakage; 8—the unit to simulate the pressure line working fluid leakage.

The data obtained by virtual modeling helps to train neural network classifiers for diagnostic systems and can save time and material resources. The SimulationX software was used to create a simulation model of the hydraulic system, Figure 4.

Subsystems 8 and 7 are used to simulate liquid leakage at the pump outlet and the gas leakage from the HPA. The value of the leakage is set by the size of the flow area of the bypass valve in the subsystem 8. A discrete 2/2 valve is used to connect the gas chamber of the HPA to the atmosphere. The safety valve adjustment error is set in the subsystem 6 by changing its cracking pressure. 

**Figure 5:**
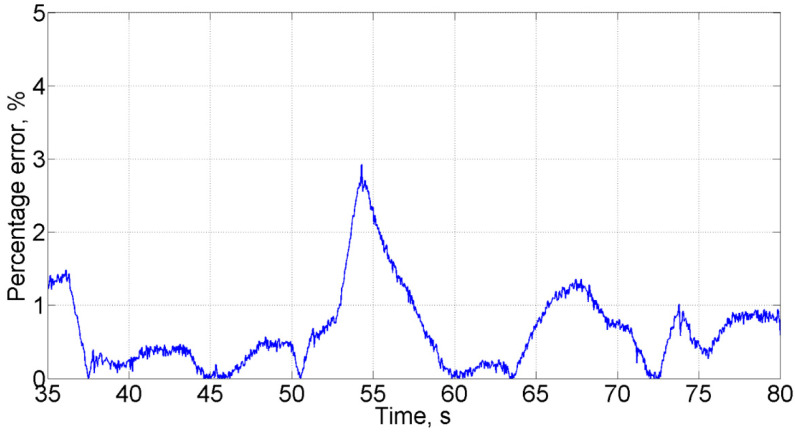
The percentage error between experimental data and simulated results of the pressure in the high-pressure line; the average deviation between the real data and the simulation does not exceed 5%.

The proportional valve 2 is supplied with a white noise signal, with characteristics identical to the physical system (Section 3.1). In order to demonstrate the equivalence of the real and the simulated data, Figure 5 shows the percentage error between experimental data and simulated results of the pressure in the high-pressure line. The data was obtained comparing the simulated system with the test bench under the same conditions after both systems reached the operating mode range, in the presence of a liquid leakage of 2.3 L/min. The analysis shows an average deviation of 1.2%, confirming the equivalence between the calculated and experimental data. For other modes, the average deviation did not exceed 5% either.

The pressure signals in the high-pressure line and the safety valve flow rate with a 100 Hz sampling rate are used as datasets for training neural network models. The simulation time for each data set is 10,000 s, which corresponds to 6666 proportional valve switches. The total number of simulated data sets for various states of the system is 121. The temperature sensor values are used only to verify that the system is in the operating mode range; in the simulation, we set a constant temperature of 50 C to the liquid. The datasets also include the corresponding control signals.

As a result, the simulated data forms an M × 3 matrix dataset that contains the pressure, the flow rate, and the control signal data. The M value is determined from a sampling rate of 100 Hz. Similar data with the same sampling rate is generated from the experimental data (Section 3.1). 

## 4. Neural Network Models Used for the IFD

### 4.1. Our Neural Network Architecture

In this paper, we present a novel model for detecting faults, a gated convolutional autoencoder-based classifier (GCAEC), with its embedding space that can be used to explain predictions. This architecture was inspired by the gated activation function in WaveNet [37] originally used for voice generation, and by the general capability of autoencoders for high-level information retrieval, which we reengineered for our fault detection.

**Figure 6:**
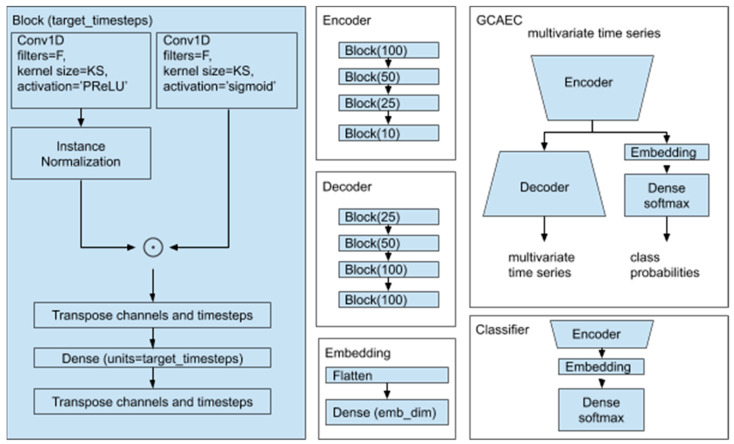
Our proposed gated convolutional autoencoder-based classifier (GCAEC) architecture.

Our proposed architecture is shown in Figure 6. While loosely based on the autoencoder architecture, our model is different. In addition to the encoder and the decoder parts, our architecture includes a jointly trained classifier. The autoencoder has gated convolution units to process the signal at each layer with shared weights between channels to perform reduction along the time axis. A multichannel shape of the signal is preserved between the layers of the autoencoder. The single flattening of the input tensor in the architecture is performed inside the embedding block. We used three hyperparameters for each block: F—the number of convolution layer filters; KS—the size of the kernel; *target_timesteps*—the number of output timesteps. 

The output of the encoder is fed into the decoder and the embedding layer. The embedding layer flattens the input and performs linear projection to the embedding space which we chose to be two-dimensional. Vectors from the embedding space then flow into the final layer of the classifier. Simultaneous training of the autoencoder with the classifier helps the classifier to account for signal characteristics and makes embedded representation of different classes separable.

The encoder and the decoder consist of the same number of blocks. Each block starts with a gated normalized one-dimensional convolution followed by the linear projection along the timestep axis. This last layer is used for learnable up- and down-sampling. The first reduction across the time axis layer determines the model parameter complexity when we vary the window size.

The gated normalized one-dimensional convolution is calculated as the element-wise product of the output of the normalized convolution layer with PReLU, and the convolution layer with sigmoid nonlinear functions. Normalization is performed across channels with *InstanceNormalization* layer.

### 4.2. Reference Neural Network Architecture

We compare the performance of our model with the performance of eight reference models: four variations of dense models, RNN, CNN, and autoencoder-based classifier models from [31] and the CNN model from [32]. 

Dense models have various number of dense layers between the normalization layer at the bottom and the dense layer with a softmax activation function at the top of the dense network. Channel normalization is performed along the batch and timestep axis by *BatchNormalization* layer. The first dense classifier consists of three dense layers with the same number of units in each layer, 50. The second one-layer dense classifier has 3 units. Dense layers receive a one-dimensional concatenation of normalized channel values since without any channel normalization, classifiers performed poorly on raw signals or could not be trained at all.

From [31] we implemented three different models: a recurrent neural network, a convolutional neural network, and an autoencoder-based classifier. We refocused these models from the original regression task to the classification task by switching the last one-output layer to “softmax” layer with N + 1 outputs. These models have the following common features: the input tensor is flattened, *tanh* nonlinear activation function is used after all layers except the top one, and they have a common structure at the top layers. The top part is the dropout layer with a dropout rate of 0.2, the dense layer has 900 units, and the dense layer has a softmax activation function which returns class probability distribution. The bottom of the RNN structure consists of two stacked bidirectional LSTM layers with 100 and 140 units. The bottom of the CNN consists of two stacked one-dimensional convolution layers with 100 and 140 kernels of size 3. An autoencoder based classifier is trained in two steps by first training the autoencoder and then using the encoder part at the bottom of the classifier. Zhao et al. autoencoder is made of the sequence of three dense layers with symmetric numbers of units—140, 280, and 140. The autoencoder input tensor is flattened and the output tensor is reshaped to match the input. 

The convolutional neural network proposed in [32] was designed to process grayscale image data. In our setup, each row represents a signal from one sensor. The network is constructed from five blocks with convolution layers. The output of the last block is flattened and fed to the dense layer with 512 units connected to two dense layers with the width equal to the number of states. Each block consists of the sequence of a convolutional layer, *BatchNormalization*, and a nonlinear activation function. Each block with convolution and dense layers, except for the last one, uses ReLU activation function, while the last dense layer uses softmax activation function.

## 5. Training Procedure

Two datasets of the training data were prepared: the simulated data only and the mix of the simulated data with the physical system data. Dataset examples are generated by a sliding window of W seconds over simulation and experimental records. Processes in the hydraulic system occur on a time scale from one millisecond to several minutes. To arrive at the optimal window size for our model to have the maximum accuracy, the minimum model size, and the minimum computational cost, we tried three window sizes: 1 s, 10 s, and 50 s. For our choice of the record sampling rate, these window sizes in seconds correspond to 100, 1000, and 5000 timesteps. The number of examples is governed by the size of records and is intentionally large for the simulated data. For our datasets, this value ranges from 10^6^ to 10^8^. For each epoch, 1 million windows were randomly sampled from multiple records to train the model.

The models are trained on raw signal values. The only exception is König and Helmi CNN model [32], trained on a normalized input per its design. We used 100 epochs and a batch size of 128.

To calculate and update weights we chose Adam [38] optimizer with β_1_ = 0.9, β_2_ = 0.98, ϵ = 10^−9^ and a learning rate λ as follows:

(2) $$\lambda \left(\mathrm{epoch}\right)=0.0003{\;\mathrm{epoch}}^{-0.5}$$

where epoch count starts with 1. 

Our training procedure minimizes two target functions. The first is the autoencoder reconstruction error, which is the mean squared error (MSE) between the input and the reconstructed signal. The second is the categorical cross-entropy error between the softmax class probability distribution and the one-hot encoding for ground-truth labels when we train the classifier. In our model we use the sum of the two:

(3) $$Loss\left(x,\;\hat{x},p\left(x\right),\hat{p}\left(x\right)\right)=\frac{1}{B}\overset{B}{\underset{i=1}{\sum }}\left({\left(x_{i}-\hat{x_{i}}\right)}^{2}-\overset{N}{\underset{c=0}{\sum }}p_{c}\left(x_{i}\right)\log\left({\hat{p}}_{c}\left(x_{i}\right)\right)\right)$$

where *x* is a set of measurements of the system state for the chosen period of time, $\hat{x}$—reconstructed measurement of the system state for the chosen period of time, *p*(*x*)—true class probability distribution, $\hat{p}\left(x\right)$—the predicted class probability distribution, and *B*—the batch size.

For performance evaluation of the classifier in the multiclass discrimination task we use the average precision, the recall, and the F_1_ measure, calculated for each class. For the anomaly detection task we report the precision, the recall, and the F_1_ measure. To compare results with [32] on ZeMA dataset [39] we also report the accuracy and the multiclass Matthews correlation coefficient.

## 6. Dataset Creation and Analysis

We collected the data from the system simulation and our physical test bench in the healthy state and with three individual faults: a leaked gas from the HPA, a continuous fluid leak, and a misconfigured safety valve. The fluid leak and misconfigured valve states were recorded for different fault parameters. Each record contains 3 channels: the pressure (bar), the flow rate (L/min), and the control signal. We recorded 274 h of the simulated data and 31 min of the experimental data from our test stand.

In our preprocessing step we converted the data from LabView and SimulationX to the standard NumPy binary file format at the chosen sampling rate of 100 Hz. Experiment equipment 5 kHz records were smoothed with a moving average filter with a 100 ms window and then down-sampled. System warmup periods were chopped off.

In our open-access repository we use a custom dataset description csv file format to select and label records. Each row contains a file path to a record, a class label, and a class name. The dataset used in our experiments consists of the following 4 states and divided into four groups which are labeled as “Healthy”, “HPA gas leakage”, “Fluid leakage”, and “Valve setting fault”.

## 7. Results and Discussion

In this section, we first present the fault detection performance of the proposed GCAEC model on our dataset for the fault detection. Then we use our GCAEC model for the fault detection using ZeMA dataset [39]. Finally, we compare these results with the performance of the reference models applied to our dataset. We used MLP classifiers as the baseline, and the RNN, the CNN, the autoencoder of [31], and the CNN model of [32] as state-of-the-art models.

All results were obtained with Tensorflow 2.4.1 software using the following hardware: Intel i9-9900KF CPU at 3.60 GHz, GeForce RTX 2080Ti, and 64 GB of RAM. 

In our experiments with GCAEC we set the number of convolutional filters F to 32 and the kernel size KS to 3. We used only pressure and flow rate channels as our experiments showed no benefit from using the control signal of the proportional valve in the fault detection task for our dataset.

**Figure 7:**
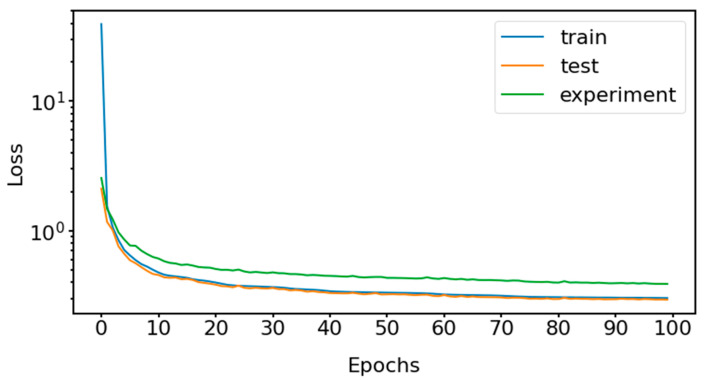
An example of the loss function optimization for GCAEC model trained for 100 epochs on the mixed dataset of samples generated by sliding a 50-s sampling window.

In Figure 7, we show how the number of epochs affects the training, test and experiment loss for our GCAEC model trained on the mixed data with a 50-s window. It illustrates that the network quickly converges within 20 epochs and then continues to slowly tune without overfitting. The data in the chart highlights the difference between simulation and experimental dataset distributions as the experiment loss is always higher than the train and test losses. This gap is four times larger when the model is trained on the simulation data only.

First, we conducted an experiment where we trained our GCAEC model on simulated and mixed datasets with different sampling window sizes. As shown in Table 1, increasing the window size yields better classification performance. For all window sizes, the most common misclassification was between the healthy state and the misconfigured safety valve state. 

Using only simulated data the model showed larger reconstruction error, and lower precision and recall. Training the model on a mixed dataset with 0.2% of experiment samples resulted in a better performance than when we used only the simulated data, as expected. The exact percentage of the experimental data was chosen by starting with 0.1% and increasing with 0.1% steps while measuring the impact. At 0.1% with a 1-s GCAEC model we observed 3% lower F_1_ values on average than at 0.2%. Training a 1-s GCAEC model on 0.3% of the experimental data in the training dataset improved the metric by less than 0.001% compared to a 0.2% mix, at which point we decided that we reached optimal results at 0.2%. The detailed analysis of how the quality changes as a function of the training dataset mix would be an interesting question to explore in the future.

**Figure 8:**
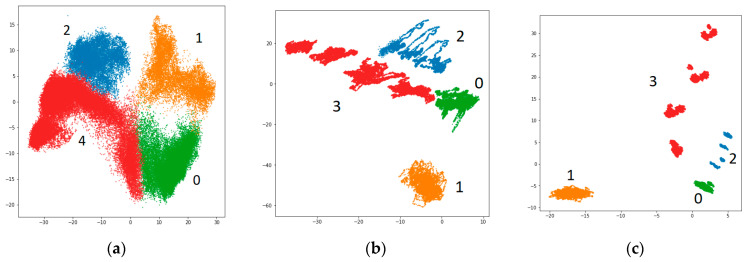
Embeddings of 4 system states with the experimental data generated by the model trained on a mixed dataset for different window sizes: (**a**) 1 s; (**b**) 10 s; (**c**) 50 s. System states legend: green (0)—a “healthy” system; orange (1)—the HPA leak; blue (2)—the fluid leak; red (3)—the valve set error.

We can conclude that a 10-s window is a good compromise between the performance and the model size. A window size also correlates with the class separation which can be seen in Figure 8. Using a window longer than 1 s made the fault degradation states to be distinguishable by the parameters in the embedding space.

The detailed performance metrics of GCAEC model trained on a 1-s window are presented in Table 2. The numbers show that using mixed data for training increases the performance for all fault types on the experimental data.

To test our proposed GCAEC architecture on different datasets, we chose ZeMA dataset [39]. This dataset contains multi-sensor data records for the condition assessment of the hydraulic test rig. The system cyclically repeats constant load cycles (of 60 s) and record sensor values from 17 sensors (the pressure, volume flow, and temperature) while the four hydraulic components (the cooler, valve, pump, and accumulator) function under different conditions. We used the same optimizer settings and the learning rate policy. For this dataset, which has fewer training examples, we increase the number of epochs to 1000. Each set of records that corresponds to one fault is split into training and testing sets according to a 7:3 ratio. We repeat the same preprocessing steps suggested in the original paper, with the only exception of channel normalization, since our model was designed to work on the raw signal values. The preprocessing step for the sensors data involved up-sampling to the maximum sampling rate of 100 Hz, and then down-sampling of the aligned dataset by averaging six consecutive values yielding 1000 timesteps per window. The resulting model had 249,553 learnable parameters. The training was performed according to [32]: four times for each fault type with predicting fault subtype. Results in Table 3 show slightly better overall performance of the GCAEC over the CNN model from [32].

Next, we looked at the performance of the chosen reference models on our dataset. We obtained comparable to GCAEC classification results with sufficiently wide reference MLP models (Table 4). The one-layer MLP that served as the baseline for neural network architecture complexity was not capable of solving the task of fault detection as shown in the last row of Table 4. From the experiment with 1-s windows we can see that to obtain a decent performance on our dataset, the dense model capacity needs to be greater than 15 thousand learnable weights. A three-layer dense model trained with a 10-s window showed slightly worse precision and recall compared to our GCAEC model.

Table 5 shows performance results for more complex reference architectures. The RNN was the slowest to train but its recall and precision were the best among reference models. The CNN model from [31] had excessive number of parameters and showed no performance benefits compared to other models. The training process of the CNN model proposed by König and Helmi [32] was unstable until we normalized input signals.

**Figure 9:**
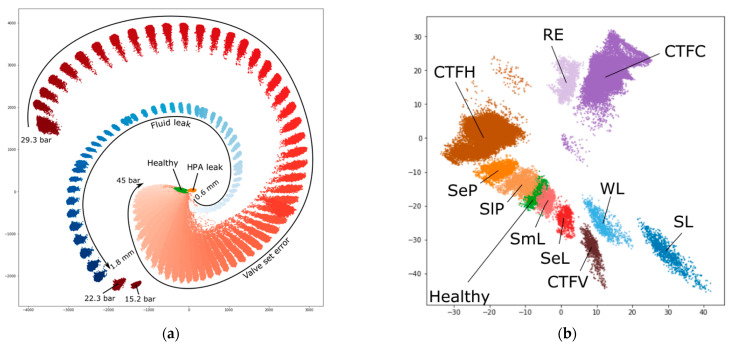
Two-dimensional embeddings of 50-s window system states returned by the GCAEC model for (**a**) our dataset and (**b**) ZeMA dataset. The axis are unitless. The legend for our dataset (**a**): green—“healthy” state; orange—the HPA leak; shades of blue—the fluid leak; shades of red—the valve set error. The visualization shows the range of “Fluid leak” and “Valve set error” fault parameters. The legend for ZeMA dataset (**b**): “CTFH”—the accumulator close to the total failure; “SeP”—the accumulator with a severely reduced pressure; “SlP”—the accumulator with a slightly reduced pressure; “SmL”—the valve with a small lag; “SeL”—the valve with a severe lag; “CTFV”—the valve close to the total failure; “WL”—the pump with weak leakage; “SL”—the pump with severe leakage; “RE”—the cooler with reduced efficiency; “CTFC”—the cooler close to the total failure.

Generated embeddings of the simulated data from the model trained to solve all-to-all timeseries classification task based on a 50-s window of pressure and fluid flow rate measurements allow to differentiate between different fault types and, also, their subtypes, which is shown in Figure 9. Cluster overlapping was consistent with the increase in classification confusion between fault subtypes. Figure 9b demonstrates the separation of mutually exclusive fault subtypes in the embedding space generated for ZeMA dataset.

## 8. Conclusions

The authors of this paper studied the performance of neural network-based algorithms in fault detection tasks in hydraulic systems, and offered a novel architecture tested on a simulated data infused with the real data obtained from the test bench hydraulic system. The proposed gated convolutional autoencoder architecture was inspired by the gated activation function used in WaveNet [37] for voice generation, and by the general capability of autoencoders for high-level information retrieval, which we reengineered for our fault detection task.

Mixing the simulated data with a small amount (0.2%) of the experimental data improved classification results on the experimental data but in some cases showed inferior performance on the simulated test set. Our model shows improved performance for the data published in [32], compared with the CNN-based classifier from [32], despite having a smaller number of learnable weights. The accuracy of 99% of correctly recognized states of the hydraulic system is obtained with a 10-s sampling window. Finally, our fault detection model provides the ability to explore the decision boundaries of the classifier in a two-dimensional embedding space, showing better explainability that many other CNN-based detection models. A larger sampling window results in better classification results and a better separation of system states in the embedding space.

Our proposed approach of mixing experimental and simulated data opens multiple directions for further research and improvements. Calculating the optimal amount of the real data infusion taking the data distribution and the noise levels into account could be a good scientific question, while studying the model changes and the impact of the hybrid approach on the time saved for model retraining in response to the hardware reconfiguration could bring this research closer to the use in the production environment based on digital twin concepts. Finally, exploring the applicability of the proposed data mixing approach and our GCAEC model for different tasks of anomaly detection, e.g., for social or biological systems, could generate a wider impact of our approach.

## Figures and Tables

**Table 1:** Average precision and recall for the classification of 4 states by our GCAEC model.

Trained on	Window Size Seconds	Tested On						# of Weights
		Simulated Data			Experimental Data			
		Precision	Recall	F_1_	Precision	Recall	F_1_	
Simulated data	1	0.9226	0.9639	0.9375	0.7463	0.8375	0.7721	84,370
Simulated data	10	0.9956	0.9956	0.9956	0.9872	0.9872	0.9829	294,070
Simulated data	50	0.9999	0.9999	0.9999	1.0000	1.0000	1.0000	1,226,070
Mixed data	1	0.9693	0.8967	0.9237	0.9525	0.9638	0.9577	84,370
Mixed data	10	0.9878	0.9873	0.9873	0.9997	0.9999	0.9998	294,070
Mixed data	50	0.9994	0.9994	0.9994	1.0000	1.0000	1.0000	1,226,070

**Table 2:** The performance metrics for each state by the GCAEC model on a 1-s sampling window.

State	Trained on Simulated or Mixed Data	Tested on					
		Simulated Data			Experimental Data		
		Precision	Recall	F_1_	Precision	Recall	F_1_
Healthy	Simulated	0.7156	0.9834	0.8284	0.7605	0.8775	0.8148
HPA leak	Simulated	0.9971	0.9834	0.9902	0.7051	0.8775	0.8148
Fluid leak	Simulated	0.9942	0.9900	0.9921	0.5536	0.8877	0.6819
Valve set error	Simulated	0.9833	0.8988	0.9391	0.9660	0.6837	0.8007
Healthy	Mixed	0.9796	0.6447	0.7776	0.9464	0.9790	0.9624
HPA leak	Mixed	0.9957	0.9530	0.9738	0.9933	0.9709	0.9820
Fluid leak	Mixed	0.9942	0.9916	0.9929	0.8924	0.9582	0.9241
Valve set error	Mixed	0.9077	0.9974	0.9505	0.9780	0.9471	0.9623

**Table 3:** Performance comparison results for the classification of 4 fault types by our GCAEC model and the CNN model [32] on ZeMA dataset for 60-s records.

Condition	GCAEC					CNN Model of [32]	
	Precision	Recall	F_1_	Accuracy%	MCC	Accuracy%	MCC
Cooler	1.0	1.0	1.0	100	1.0	99.6	0.992
Valve	1.0	1.0	1.0	100	1.0	100	1.0
Pump	0.993	0.994	0.9935	99.54	0.992	96.9	0.914
Accumulator	0.975	0.971	0.9730	97.73	0.968	98.2	0.947

**Table 4:** Performance metrics for classification of 4 system states by different NN architectures.

Layers in MLP Model	Window	Simulated or Mixed Data	Tested On						# of Weights
			Simulated Data			Experimental Data			
			Precision	Recall	F_1_	Precision	Recall	F_1_	
Three	10 s	Simulated	0.9982	0.9859	0.9920	0.9581	0.9723	0.9651	105,362
Three	10 s	Mixed	0.9979	0.9860	0.9919	0.9988	0.9991	0.9989	105,362
Three	1 s	Simulated	0.9844	0.8214	0.8955	0.8407	0.8360	0.8383	15,362
One	1 s	Mixed	0.6571	0.5174	0.5789	0.4304	0.4961	0.4609	627

**Table 5:** Performance metrics for the classification of 4 system states by reference NN architectures. Models are trained with 10-s windows sampled from a mixed dataset.

Architecture	Tested on						# of Weights
	Simulated Data			Experimental Data			
	Precision	Recall	F_1_	Precision	Recall	F_1_	
Zhao et al. RNN [31]	0.994	0.990	0.992	1.000	1.000	1.000	720,824
Zhao et al. CNN [31]	0.998	0.981	0.989	0.995	0.998	0.996	62,795,344
Zhao et al. Autoencoder based classifier [31]	0.995	0.983	0.989	1.000	1.000	1.000	344,752
König, Helmi CNN [32]	0.988	0.992	0.990	0.999	0.998	0.998	358,328
GCAEC	0.999	0.999	0.999	0.999	0.999	0.999	294,070

## Data Availability

The source code, the simulated and physical datasets used in this paper are available at https://gitlab.com/protsenkovi/efd_nn (access on 25 June 2021).
